# Supplementary material for: Pyruvate Dehydrogenase Kinase 1 inhibition mediated oxidative phosphorylation enhancement in cartilage promotes osteoarthritis progression
Source: BMC Musculoskelet Disord. 2023 Jul 20;24:597. doi: 10.1186/s12891-023-06585-6 (PMC10357736; doi:10.1186/s12891-023-06585-6)
Supplement: Supplementary file 1 — Additional file 1. [file 12891_2023_6585_MOESM1_ESM.zip › MF.Gsea.1653627316242/GOMF_HYDROLASE_ACTIVITY_ACTING_ON_GLYCOSYL_BONDS.html]

Details for gene set GOMF\_HYDROLASE\_ACTIVITY\_ACTING\_ON\_GLYCOSYL\_BONDS[GSEA]

|  || Dataset | 55457\_collapsed\_to\_symbols.cls.cls #OA\_versus\_normal.cls.cls #OA\_versus\_normal\_repos |
| Phenotype | cls.cls#OA\_versus\_normal\_repos |
| Upregulated in class | OA |
| GeneSet | GOMF\_HYDROLASE\_ACTIVITY\_ACTING\_ON\_GLYCOSYL\_BONDS |
| Enrichment Score (ES) | 0.2888078 |
| Normalized Enrichment Score (NES) | 1.344577 |
| Nominal p-value | 0.04010025 |
| FDR q-value | 0.35887802 |
| FWER p-Value | 1.0 |
Table: GSEA Results Summary

  

Fig 1: Enrichment plot: GOMF\_HYDROLASE\_ACTIVITY\_ACTING\_ON\_GLYCOSYL\_BONDS      
 Profile of the Running ES Score & Positions of GeneSet Members on the Rank Ordered List

  

| SYMBOL | TITLE | RANK IN GENE LIST | RANK METRIC SCORE | RUNNING ES | CORE ENRICHMENT || 1 | TLR7 | toll like receptor 7 [Source:HGNC Symbol;Acc:HGNC:15631] | 61 | 0.971 | 0.0211 | Yes |
| 2 | TLR3 | toll like receptor 3 [Source:HGNC Symbol;Acc:HGNC:11849] | 175 | 0.837 | 0.0345 | Yes |
| 3 | SPAM1 | sperm adhesion molecule 1 [Source:HGNC Symbol;Acc:HGNC:11217] | 180 | 0.831 | 0.0564 | Yes |
| 4 | CLN5 | CLN5 intracellular trafficking protein [Source:HGNC Symbol;Acc:HGNC:2076] | 248 | 0.785 | 0.0720 | Yes |
| 5 | MPG | N-methylpurine DNA glycosylase [Source:HGNC Symbol;Acc:HGNC:7211] | 277 | 0.768 | 0.0903 | Yes |
| 6 | GLB1 | galactosidase beta 1 [Source:HGNC Symbol;Acc:HGNC:4298] | 337 | 0.731 | 0.1051 | Yes |
| 7 | ABHD10 | "abhydrolase domain containing 10, depalmitoylase [Source:HGNC Symbol;Acc:HGNC:25656]" | 437 | 0.690 | 0.1157 | Yes |
| 8 | OGG1 | 8-oxoguanine DNA glycosylase [Source:HGNC Symbol;Acc:HGNC:8125] | 453 | 0.681 | 0.1327 | Yes |
| 9 | GALC | galactosylceramidase [Source:HGNC Symbol;Acc:HGNC:4115] | 478 | 0.669 | 0.1486 | Yes |
| 10 | DNPH1 | 2'-deoxynucleoside 5'-phosphate N-hydrolase 1 [Source:HGNC Symbol;Acc:HGNC:21218] | 719 | 0.589 | 0.1456 | Yes |
| 11 | BANK1 | B cell scaffold protein with ankyrin repeats 1 [Source:HGNC Symbol;Acc:HGNC:18233] | 802 | 0.568 | 0.1543 | Yes |
| 12 | GUSB | glucuronidase beta [Source:HGNC Symbol;Acc:HGNC:4696] | 904 | 0.541 | 0.1608 | Yes |
| 13 | MANEA | mannosidase endo-alpha [Source:HGNC Symbol;Acc:HGNC:21072] | 950 | 0.528 | 0.1714 | Yes |
| 14 | HYAL1 | hyaluronidase 1 [Source:HGNC Symbol;Acc:HGNC:5320] | 997 | 0.518 | 0.1815 | Yes |
| 15 | SMUG1 | single-strand-selective monofunctional uracil-DNA glycosylase 1 [Source:HGNC Symbol;Acc:HGNC:17148] | 1068 | 0.501 | 0.1894 | Yes |
| 16 | MAN2A1 | mannosidase alpha class 2A member 1 [Source:HGNC Symbol;Acc:HGNC:6824] | 1069 | 0.501 | 0.2028 | Yes |
| 17 | GAA | alpha glucosidase [Source:HGNC Symbol;Acc:HGNC:4065] | 1149 | 0.486 | 0.2095 | Yes |
| 18 | SMPD1 | sphingomyelin phosphodiesterase 1 [Source:HGNC Symbol;Acc:HGNC:11120] | 1230 | 0.471 | 0.2158 | Yes |
| 19 | GANAB | glucosidase II alpha subunit [Source:HGNC Symbol;Acc:HGNC:4138] | 1267 | 0.464 | 0.2254 | Yes |
| 20 | MAN1C1 | mannosidase alpha class 1C member 1 [Source:HGNC Symbol;Acc:HGNC:19080] | 1275 | 0.463 | 0.2371 | Yes |
| 21 | CTBS | chitobiase [Source:HGNC Symbol;Acc:HGNC:2496] | 1333 | 0.453 | 0.2447 | Yes |
| 22 | HEXB | hexosaminidase subunit beta [Source:HGNC Symbol;Acc:HGNC:4879] | 1434 | 0.438 | 0.2486 | Yes |
| 23 | FUCA1 | alpha-L-fucosidase 1 [Source:HGNC Symbol;Acc:HGNC:4006] | 1506 | 0.428 | 0.2544 | Yes |
| 24 | GLB1L | galactosidase beta 1 like [Source:HGNC Symbol;Acc:HGNC:28129] | 1544 | 0.421 | 0.2627 | Yes |
| 25 | PCNA | proliferating cell nuclear antigen [Source:HGNC Symbol;Acc:HGNC:8729] | 1557 | 0.419 | 0.2730 | Yes |
| 26 | NAGPA | N-acetylglucosamine-1-phosphodiester alpha-N-acetylglucosaminidase [Source:HGNC Symbol;Acc:HGNC:17378] | 1608 | 0.412 | 0.2800 | Yes |
| 27 | NAGA | alpha-N-acetylgalactosaminidase [Source:HGNC Symbol;Acc:HGNC:7631] | 1674 | 0.402 | 0.2857 | Yes |
| 28 | GBA3 | glucosylceramidase beta 3 (gene/pseudogene) [Source:HGNC Symbol;Acc:HGNC:19069] | 1767 | 0.388 | 0.2888 | Yes |
| 29 | EDEM2 | ER degradation enhancing alpha-mannosidase like protein 2 [Source:HGNC Symbol;Acc:HGNC:15877] | 2076 | 0.353 | 0.2741 | No |
| 30 | GM2A | GM2 ganglioside activator [Source:HGNC Symbol;Acc:HGNC:4367] | 2299 | 0.327 | 0.2655 | No |
| 31 | BST1 | bone marrow stromal cell antigen 1 [Source:HGNC Symbol;Acc:HGNC:1118] | 2319 | 0.324 | 0.2726 | No |
| 32 | HPSE | heparanase [Source:HGNC Symbol;Acc:HGNC:5164] | 2360 | 0.319 | 0.2780 | No |
| 33 | CD38 | CD38 molecule [Source:HGNC Symbol;Acc:HGNC:1667] | 2416 | 0.312 | 0.2820 | No |
| 34 | OARD1 | O-acyl-ADP-ribose deacylase 1 [Source:HGNC Symbol;Acc:HGNC:21257] | 2713 | 0.278 | 0.2663 | No |
| 35 | NAGLU | N-acetyl-alpha-glucosaminidase [Source:HGNC Symbol;Acc:HGNC:7632] | 2767 | 0.273 | 0.2694 | No |
| 36 | HEXA | hexosaminidase subunit alpha [Source:HGNC Symbol;Acc:HGNC:4878] | 3025 | 0.247 | 0.2559 | No |
| 37 | TLR1 | toll like receptor 1 [Source:HGNC Symbol;Acc:HGNC:11847] | 3049 | 0.245 | 0.2606 | No |
| 38 | SIGIRR | single Ig and TIR domain containing [Source:HGNC Symbol;Acc:HGNC:30575] | 3109 | 0.239 | 0.2624 | No |
| 39 | NTHL1 | nth like DNA glycosylase 1 [Source:HGNC Symbol;Acc:HGNC:8028] | 3219 | 0.228 | 0.2599 | No |
| 40 | TLR5 | toll like receptor 5 [Source:HGNC Symbol;Acc:HGNC:11851] | 3261 | 0.225 | 0.2627 | No |
| 41 | HPSE2 | heparanase 2 (inactive) [Source:HGNC Symbol;Acc:HGNC:18374] | 3318 | 0.219 | 0.2642 | No |
| 42 | GBA | glucosylceramidase beta [Source:HGNC Symbol;Acc:HGNC:4177] | 3327 | 0.219 | 0.2694 | No |
| 43 | MAN1B1 | mannosidase alpha class 1B member 1 [Source:HGNC Symbol;Acc:HGNC:6823] | 3447 | 0.207 | 0.2656 | No |
| 44 | AGL | "amylo-alpha-1, 6-glucosidase, 4-alpha-glucanotransferase [Source:HGNC Symbol;Acc:HGNC:321]" | 3537 | 0.199 | 0.2639 | No |
| 45 | MAN2B1 | mannosidase alpha class 2B member 1 [Source:HGNC Symbol;Acc:HGNC:6826] | 3577 | 0.196 | 0.2661 | No |
| 46 | TLR8 | toll like receptor 8 [Source:HGNC Symbol;Acc:HGNC:15632] | 3579 | 0.196 | 0.2712 | No |
| 47 | CEMIP | cell migration inducing hyaluronidase 1 [Source:HGNC Symbol;Acc:HGNC:29213] | 3747 | 0.182 | 0.2630 | No |
| 48 | CHI3L2 | chitinase 3 like 2 [Source:HGNC Symbol;Acc:HGNC:1933] | 3821 | 0.174 | 0.2620 | No |
| 49 | NEIL3 | nei like DNA glycosylase 3 [Source:HGNC Symbol;Acc:HGNC:24573] | 4006 | 0.156 | 0.2517 | No |
| 50 | SI | sucrase-isomaltase [Source:HGNC Symbol;Acc:HGNC:10856] | 4021 | 0.154 | 0.2547 | No |
| 51 | SARM1 | sterile alpha and TIR motif containing 1 [Source:HGNC Symbol;Acc:HGNC:17074] | 4083 | 0.148 | 0.2539 | No |
| 52 | GLA | galactosidase alpha [Source:HGNC Symbol;Acc:HGNC:4296] | 4172 | 0.140 | 0.2507 | No |
| 53 | MAN1A2 | mannosidase alpha class 1A member 2 [Source:HGNC Symbol;Acc:HGNC:6822] | 4272 | 0.131 | 0.2465 | No |
| 54 | NEU1 | neuraminidase 1 [Source:HGNC Symbol;Acc:HGNC:7758] | 4486 | 0.114 | 0.2329 | No |
| 55 | MAN2B2 | mannosidase alpha class 2B member 2 [Source:HGNC Symbol;Acc:HGNC:29623] | 4548 | 0.109 | 0.2310 | No |
| 56 | KL | klotho [Source:HGNC Symbol;Acc:HGNC:6344] | 4901 | 0.080 | 0.2056 | No |
| 57 | LYZL6 | lysozyme like 6 [Source:HGNC Symbol;Acc:HGNC:29614] | 4929 | 0.078 | 0.2056 | No |
| 58 | IDUA | alpha-L-iduronidase [Source:HGNC Symbol;Acc:HGNC:5391] | 5002 | 0.073 | 0.2019 | No |
| 59 | MACROD1 | mono-ADP ribosylhydrolase 1 [Source:HGNC Symbol;Acc:HGNC:29598] | 5008 | 0.072 | 0.2034 | No |
| 60 | MUTYH | mutY DNA glycosylase [Source:HGNC Symbol;Acc:HGNC:7527] | 5252 | 0.052 | 0.1858 | No |
| 61 | CHI3L1 | chitinase 3 like 1 [Source:HGNC Symbol;Acc:HGNC:1932] | 5598 | 0.024 | 0.1595 | No |
| 62 | EDEM1 | ER degradation enhancing alpha-mannosidase like protein 1 [Source:HGNC Symbol;Acc:HGNC:18967] | 5633 | 0.021 | 0.1574 | No |
| 63 | NEU2 | neuraminidase 2 [Source:HGNC Symbol;Acc:HGNC:7759] | 5775 | 0.010 | 0.1466 | No |
| 64 | EDEM3 | ER degradation enhancing alpha-mannosidase like protein 3 [Source:HGNC Symbol;Acc:HGNC:16787] | 6073 | -0.010 | 0.1237 | No |
| 65 | UNG | uracil DNA glycosylase [Source:HGNC Symbol;Acc:HGNC:12572] | 6078 | -0.011 | 0.1236 | No |
| 66 | MANBA | mannosidase beta [Source:HGNC Symbol;Acc:HGNC:6831] | 6373 | -0.033 | 0.1015 | No |
| 67 | MYD88 | MYD88 innate immune signal transduction adaptor [Source:HGNC Symbol;Acc:HGNC:7562] | 6638 | -0.052 | 0.0823 | No |
| 68 | OGA | O-GlcNAcase [Source:HGNC Symbol;Acc:HGNC:7056] | 7086 | -0.086 | 0.0497 | No |
| 69 | SMPDL3B | sphingomyelin phosphodiesterase acid like 3B [Source:HGNC Symbol;Acc:HGNC:21416] | 7293 | -0.102 | 0.0363 | No |
| 70 | GLB1L2 | galactosidase beta 1 like 2 [Source:HGNC Symbol;Acc:HGNC:25129] | 7341 | -0.105 | 0.0354 | No |
| 71 | TLR4 | toll like receptor 4 [Source:HGNC Symbol;Acc:HGNC:11850] | 7703 | -0.134 | 0.0107 | No |
| 72 | TLR2 | toll like receptor 2 [Source:HGNC Symbol;Acc:HGNC:11848] | 7991 | -0.154 | -0.0076 | No |
| 73 | CHIT1 | chitinase 1 [Source:HGNC Symbol;Acc:HGNC:1936] | 8207 | -0.172 | -0.0198 | No |
| 74 | HYAL4 | hyaluronidase 4 [Source:HGNC Symbol;Acc:HGNC:5323] | 8209 | -0.172 | -0.0153 | No |
| 75 | IL1RAPL2 | interleukin 1 receptor accessory protein like 2 [Source:HGNC Symbol;Acc:HGNC:5997] | 8437 | -0.189 | -0.0280 | No |
| 76 | LCT | lactase [Source:HGNC Symbol;Acc:HGNC:6530] | 8444 | -0.189 | -0.0235 | No |
| 77 | APEX1 | apurinic/apyrimidinic endodeoxyribonuclease 1 [Source:HGNC Symbol;Acc:HGNC:587] | 8767 | -0.213 | -0.0429 | No |
| 78 | MAN2C1 | mannosidase alpha class 2C member 1 [Source:HGNC Symbol;Acc:HGNC:6827] | 9342 | -0.259 | -0.0809 | No |
| 79 | NEU3 | neuraminidase 3 [Source:HGNC Symbol;Acc:HGNC:7760] | 9348 | -0.259 | -0.0744 | No |
| 80 | IL1RAP | interleukin 1 receptor accessory protein [Source:HGNC Symbol;Acc:HGNC:5995] | 9395 | -0.263 | -0.0710 | No |
| 81 | TICAM1 | toll like receptor adaptor molecule 1 [Source:HGNC Symbol;Acc:HGNC:18348] | 9569 | -0.280 | -0.0771 | No |
| 82 | CHIA | chitinase acidic [Source:HGNC Symbol;Acc:HGNC:17432] | 9641 | -0.287 | -0.0750 | No |
| 83 | IL18RAP | interleukin 18 receptor accessory protein [Source:HGNC Symbol;Acc:HGNC:5989] | 9832 | -0.304 | -0.0817 | No |
| 84 | IL1RL1 | interleukin 1 receptor like 1 [Source:HGNC Symbol;Acc:HGNC:5998] | 9840 | -0.305 | -0.0742 | No |
| 85 | TREH | trehalase [Source:HGNC Symbol;Acc:HGNC:12266] | 9910 | -0.313 | -0.0712 | No |
| 86 | IL1RAPL1 | interleukin 1 receptor accessory protein like 1 [Source:HGNC Symbol;Acc:HGNC:5996] | 10020 | -0.323 | -0.0712 | No |
| 87 | MGAM | maltase-glucoamylase [Source:HGNC Symbol;Acc:HGNC:7043] | 10106 | -0.331 | -0.0690 | No |
| 88 | MAN1A1 | mannosidase alpha class 1A member 1 [Source:HGNC Symbol;Acc:HGNC:6821] | 10207 | -0.341 | -0.0677 | No |
| 89 | GBE1 | "1,4-alpha-glucan branching enzyme 1 [Source:HGNC Symbol;Acc:HGNC:4180]" | 10356 | -0.357 | -0.0698 | No |
| 90 | HYAL2 | hyaluronidase 2 [Source:HGNC Symbol;Acc:HGNC:5321] | 10420 | -0.363 | -0.0650 | No |
| 91 | LYZ | lysozyme [Source:HGNC Symbol;Acc:HGNC:6740] | 10483 | -0.371 | -0.0600 | No |
| 92 | MOGS | mannosyl-oligosaccharide glucosidase [Source:HGNC Symbol;Acc:HGNC:24862] | 10630 | -0.386 | -0.0611 | No |
| 93 | TDG | thymine DNA glycosylase [Source:HGNC Symbol;Acc:HGNC:11700] | 10728 | -0.396 | -0.0582 | No |
| 94 | OVGP1 | oviductal glycoprotein 1 [Source:HGNC Symbol;Acc:HGNC:8524] | 10740 | -0.398 | -0.0484 | No |
| 95 | IL18R1 | interleukin 18 receptor 1 [Source:HGNC Symbol;Acc:HGNC:5988] | 10822 | -0.407 | -0.0439 | No |
| 96 | TLR6 | toll like receptor 6 [Source:HGNC Symbol;Acc:HGNC:16711] | 10941 | -0.423 | -0.0419 | No |
| 97 | NEIL1 | nei like DNA glycosylase 1 [Source:HGNC Symbol;Acc:HGNC:18448] | 10963 | -0.425 | -0.0322 | No |
| 98 | PGGHG | protein-glucosylgalactosylhydroxylysine glucosidase [Source:HGNC Symbol;Acc:HGNC:26210] | 11033 | -0.434 | -0.0260 | No |
| 99 | ENGASE | endo-beta-N-acetylglucosaminidase [Source:HGNC Symbol;Acc:HGNC:24622] | 11056 | -0.439 | -0.0160 | No |
| 100 | MBD4 | "methyl-CpG binding domain 4, DNA glycosylase [Source:HGNC Symbol;Acc:HGNC:6919]" | 11114 | -0.445 | -0.0086 | No |
| 101 | RPS3 | ribosomal protein S3 [Source:HGNC Symbol;Acc:HGNC:10420] | 11245 | -0.463 | -0.0065 | No |
| 102 | AMY1A | amylase alpha 1A [Source:HGNC Symbol;Acc:HGNC:474] | 11681 | -0.529 | -0.0264 | No |
| 103 | MAN2A2 | mannosidase alpha class 2A member 2 [Source:HGNC Symbol;Acc:HGNC:6825] | 11772 | -0.545 | -0.0189 | No |
| 104 | PARG | poly(ADP-ribose) glycohydrolase [Source:HGNC Symbol;Acc:HGNC:8605] | 11786 | -0.547 | -0.0054 | No |
| 105 | CEMIP2 | cell migration inducing hyaluronidase 2 [Source:HGNC Symbol;Acc:HGNC:11869] | 11971 | -0.588 | -0.0041 | No |
| 106 | ADPRH | ADP-ribosylarginine hydrolase [Source:HGNC Symbol;Acc:HGNC:269] | 12277 | -0.670 | -0.0101 | No |
| 107 | IL1R1 | interleukin 1 receptor type 1 [Source:HGNC Symbol;Acc:HGNC:5993] | 12419 | -0.713 | -0.0021 | No |
| 108 | IL1RL2 | interleukin 1 receptor like 2 [Source:HGNC Symbol;Acc:HGNC:5999] | 12435 | -0.719 | 0.0158 | No |
| 109 | GNE | glucosamine (UDP-N-acetyl)-2-epimerase/N-acetylmannosamine kinase [Source:HGNC Symbol;Acc:HGNC:23657] | 12538 | -0.767 | 0.0283 | No |
Table: GSEA details [plain text format]

  

Fig 2: GOMF\_HYDROLASE\_ACTIVITY\_ACTING\_ON\_GLYCOSYL\_BONDS      
 Blue-Pink O' Gram in the Space of the Analyzed GeneSet

  

Fig 3: GOMF\_HYDROLASE\_ACTIVITY\_ACTING\_ON\_GLYCOSYL\_BONDS: Random ES distribution      
 Gene set null distribution of ES for **GOMF\_HYDROLASE\_ACTIVITY\_ACTING\_ON\_GLYCOSYL\_BONDS**
